# Correspondence

**Published:** 1887-07

**Authors:** A. F. Randell

**Affiliations:** Port Huron, Michigan


### CORRESPONDENCE.

PORT HURON, MICHIGAN, May 30th, 1887.

DEAR EDITOR:—*In re Ice Suppositories*, the *Massachusetts Medical Journal* is not putting forth any new thing, as the inclosed, copied from the *Canada Lancet*, of November, 1871, shows:

"*Ice in the Rectum in Retention of Urine.*—Dr. Casenave has for the last twenty years used ice in retention of urine, and has never failed in giving relief. He introduces into the rectum a piece of ice of the form of an elongated oval and about the size of a chestnut, which he pushes up beyond the sphincters, and renews every two hours.

"Almost always in an hour and a half urethral spasm ceases, a certain quantity of urine is passed, and the bladder is emptied without effort by the patient.

"If, in rare and exceptional cases, this does not take place, he, besides this, places ice from the anus to the end of the penis until the urine flows, which it infallibly does. Where prostatic hypertrophy causes the difficulty, the good effects *of the ice are longer coming on.*" A. F. RANDELL.

